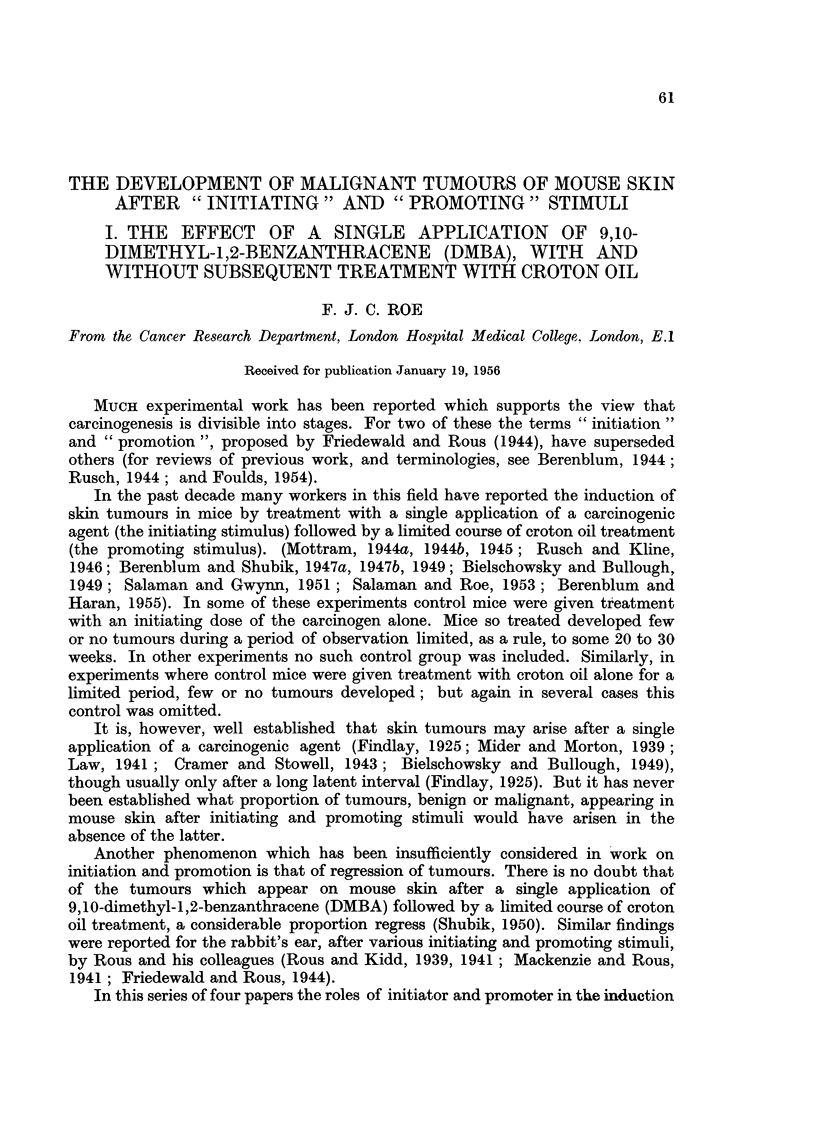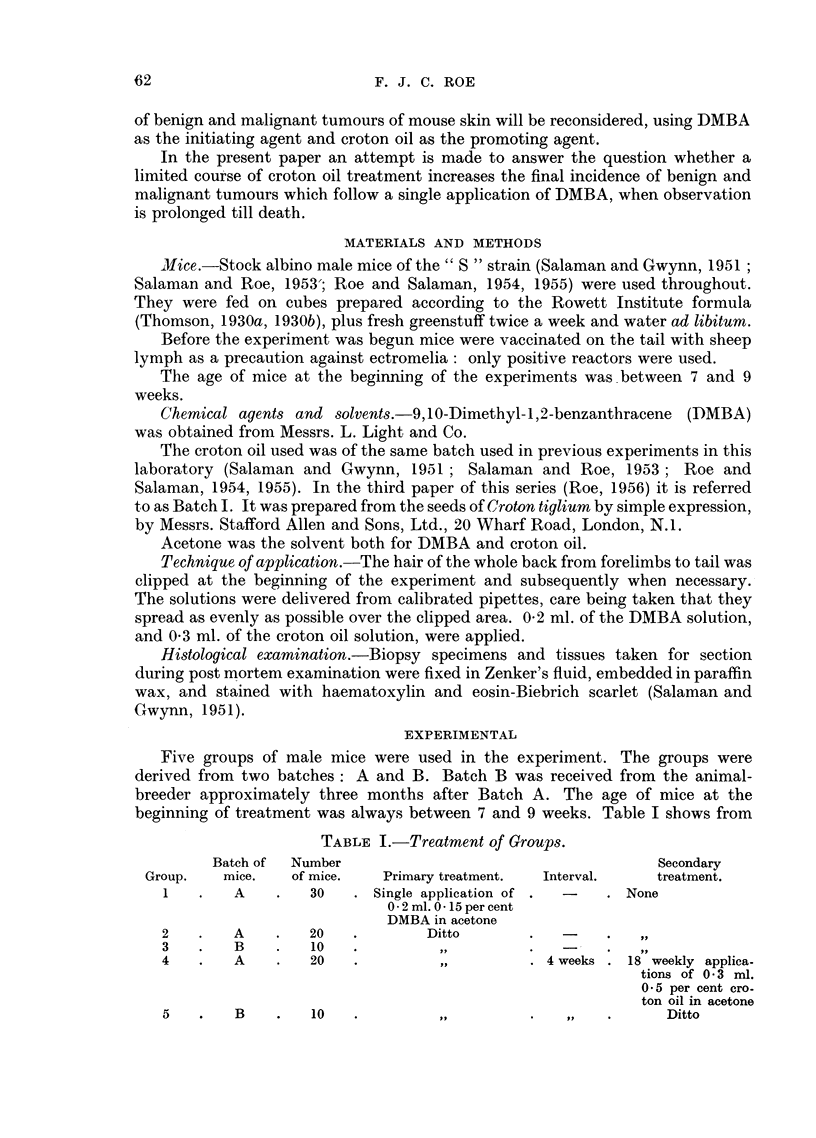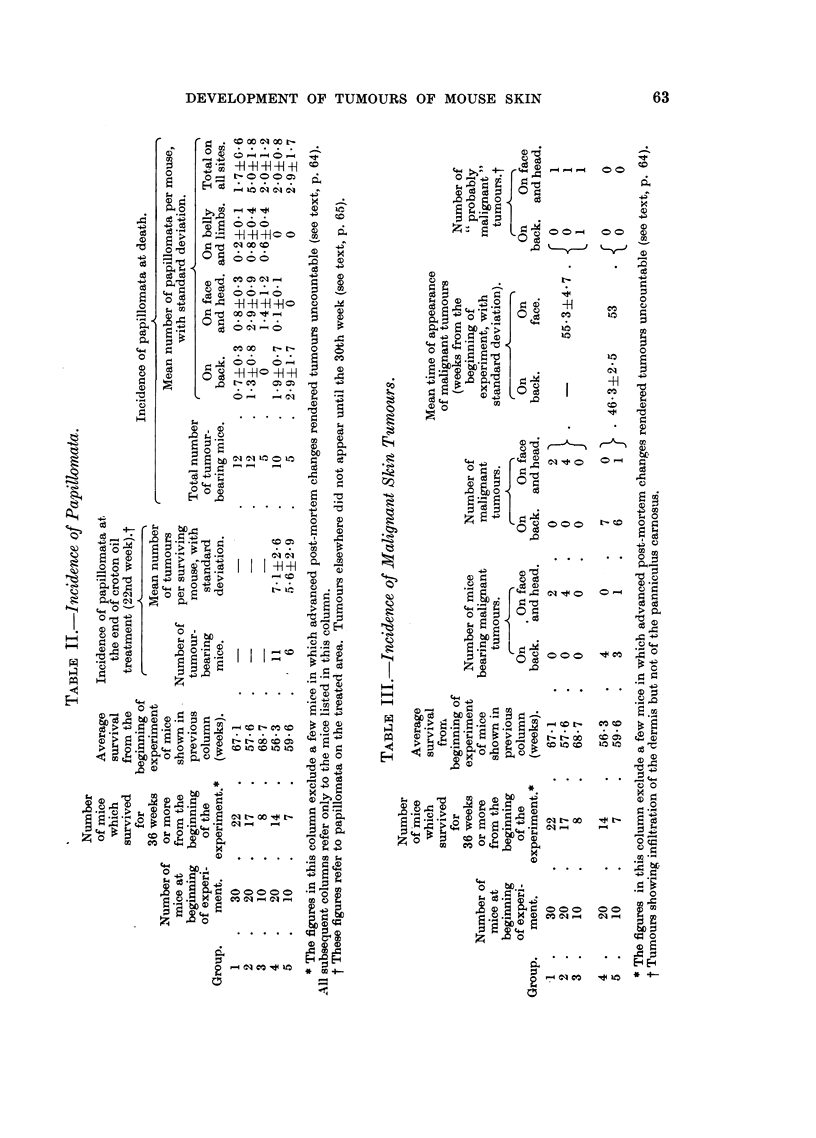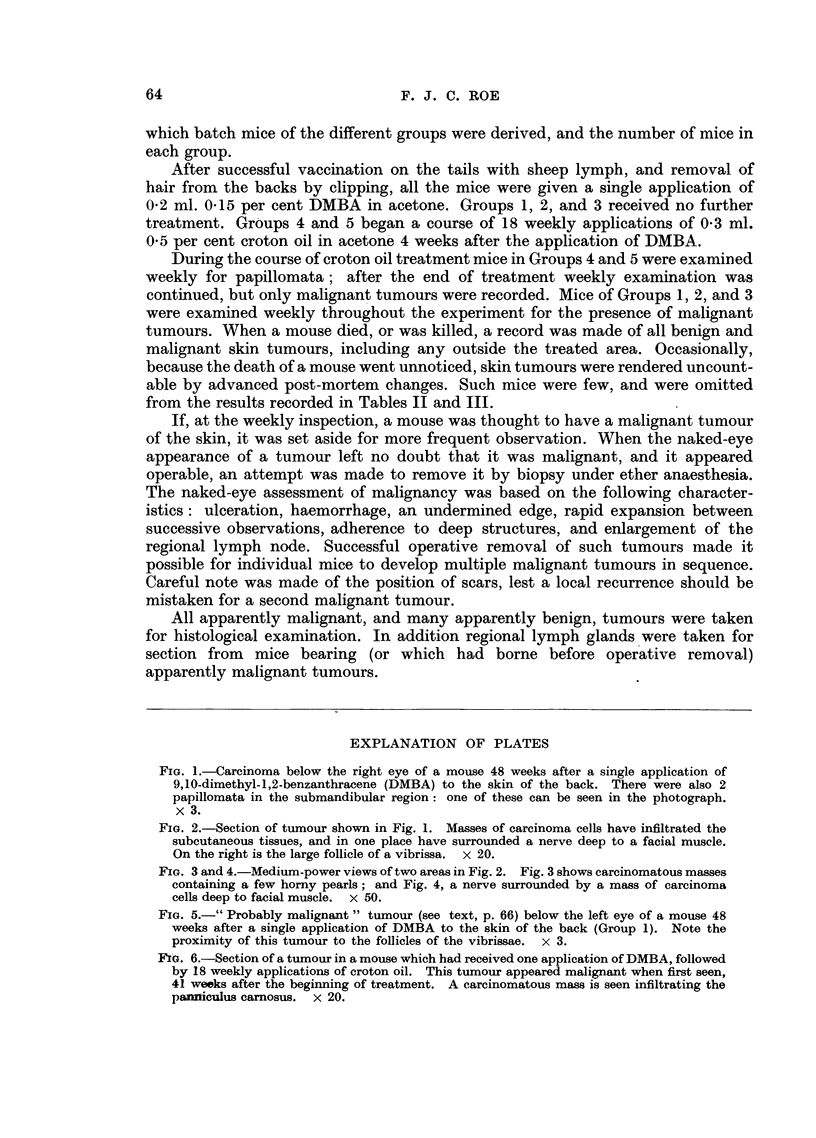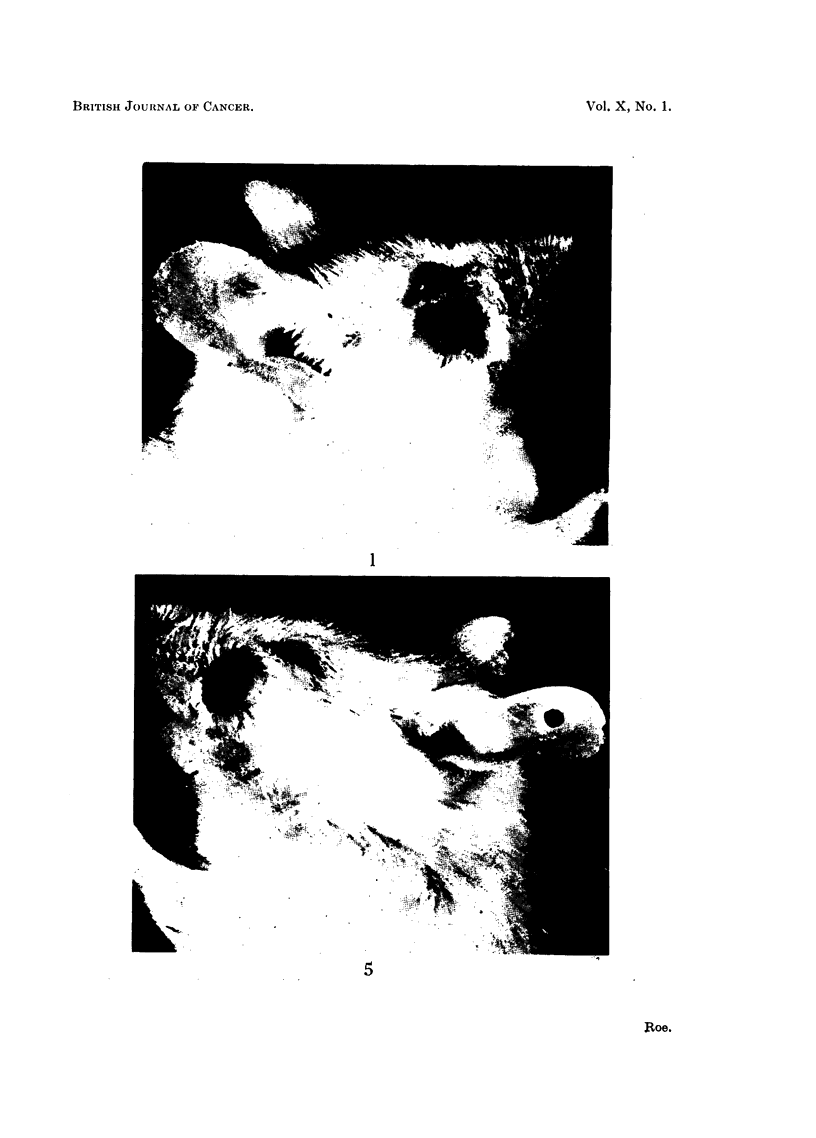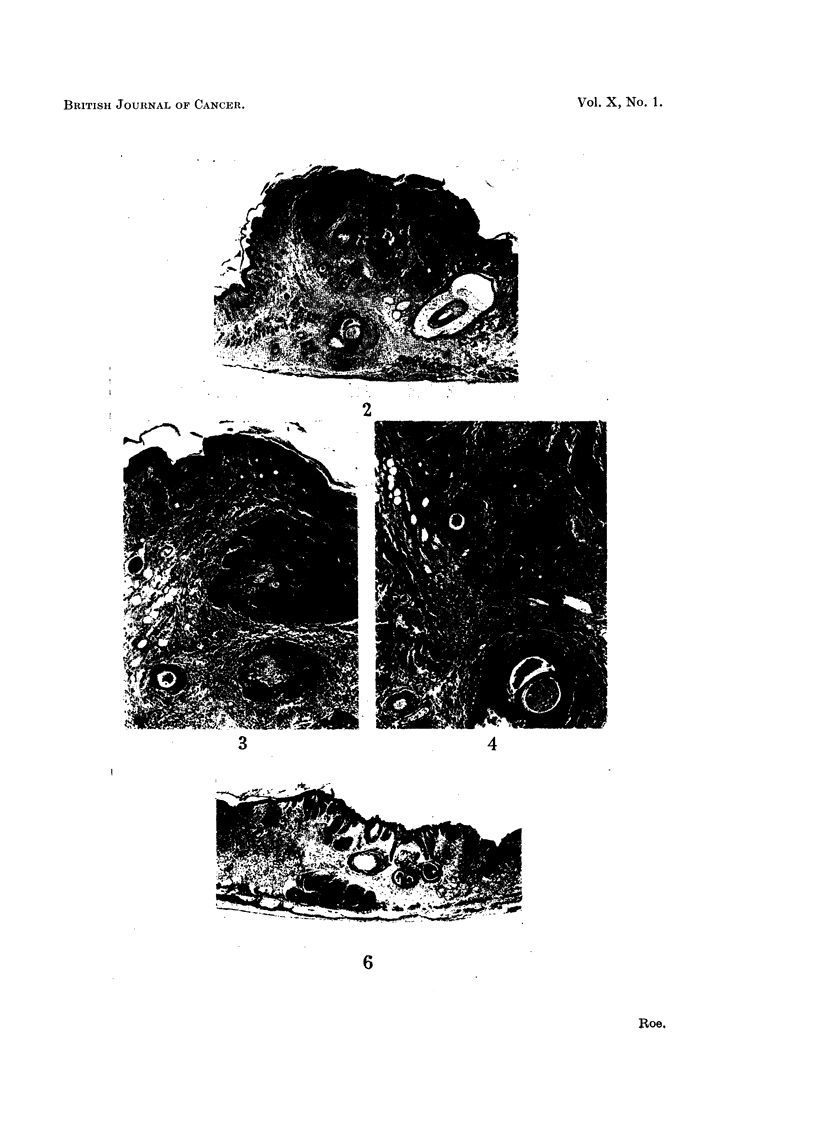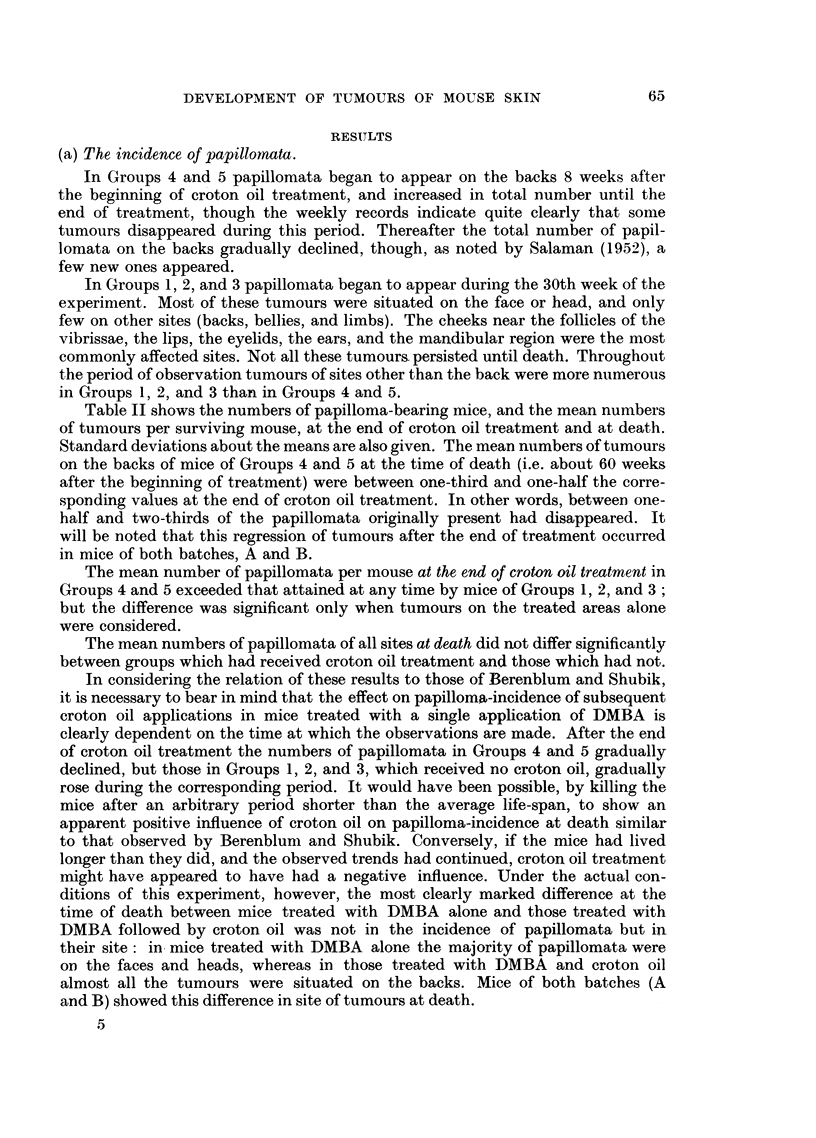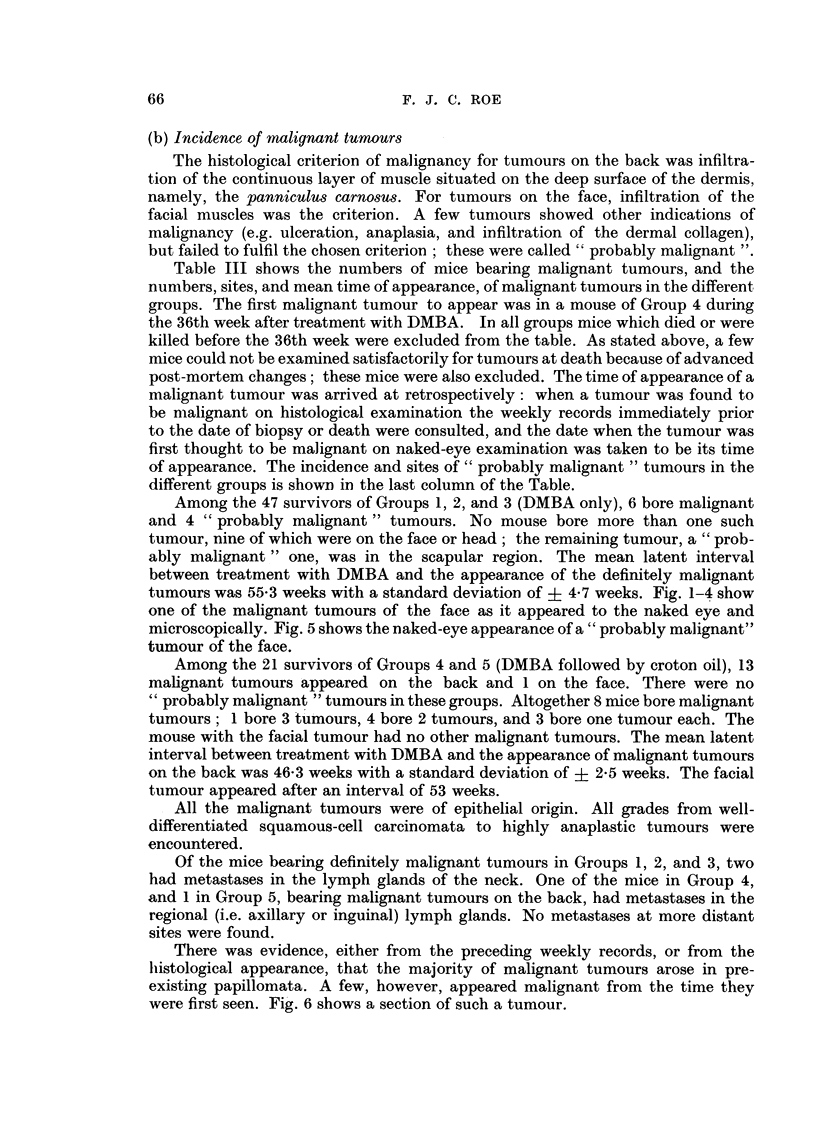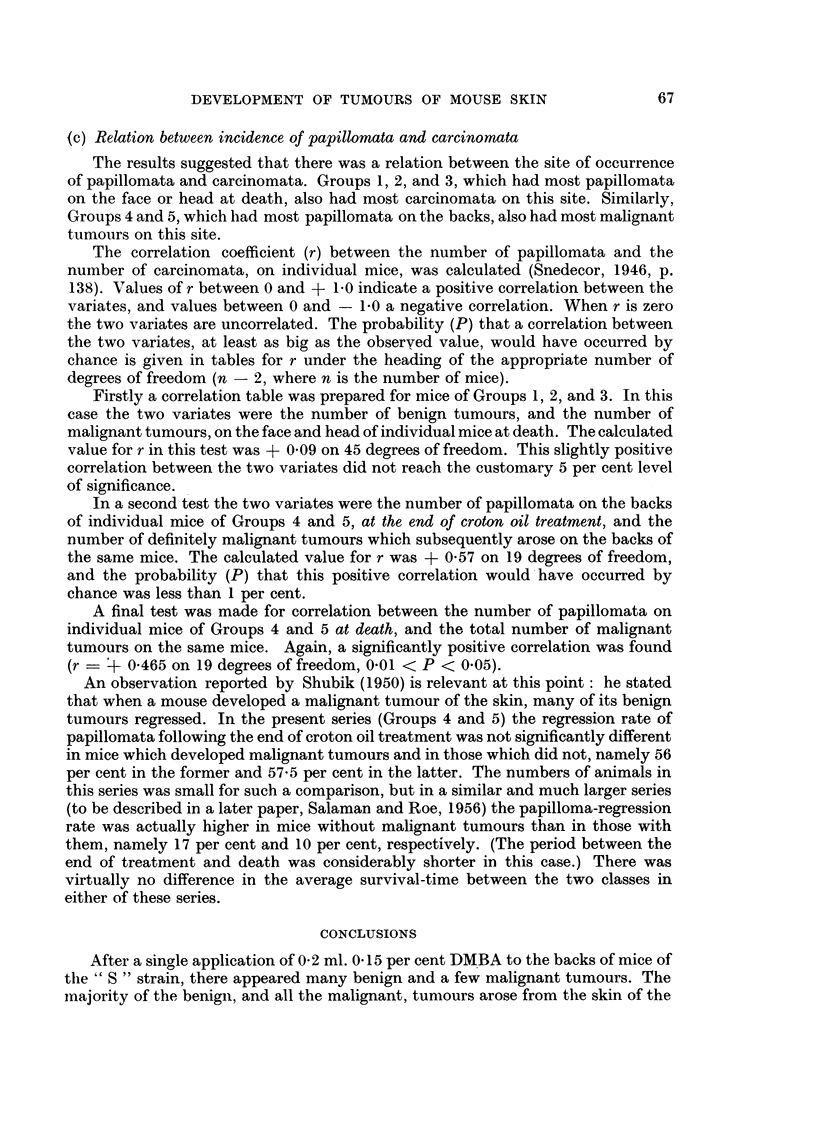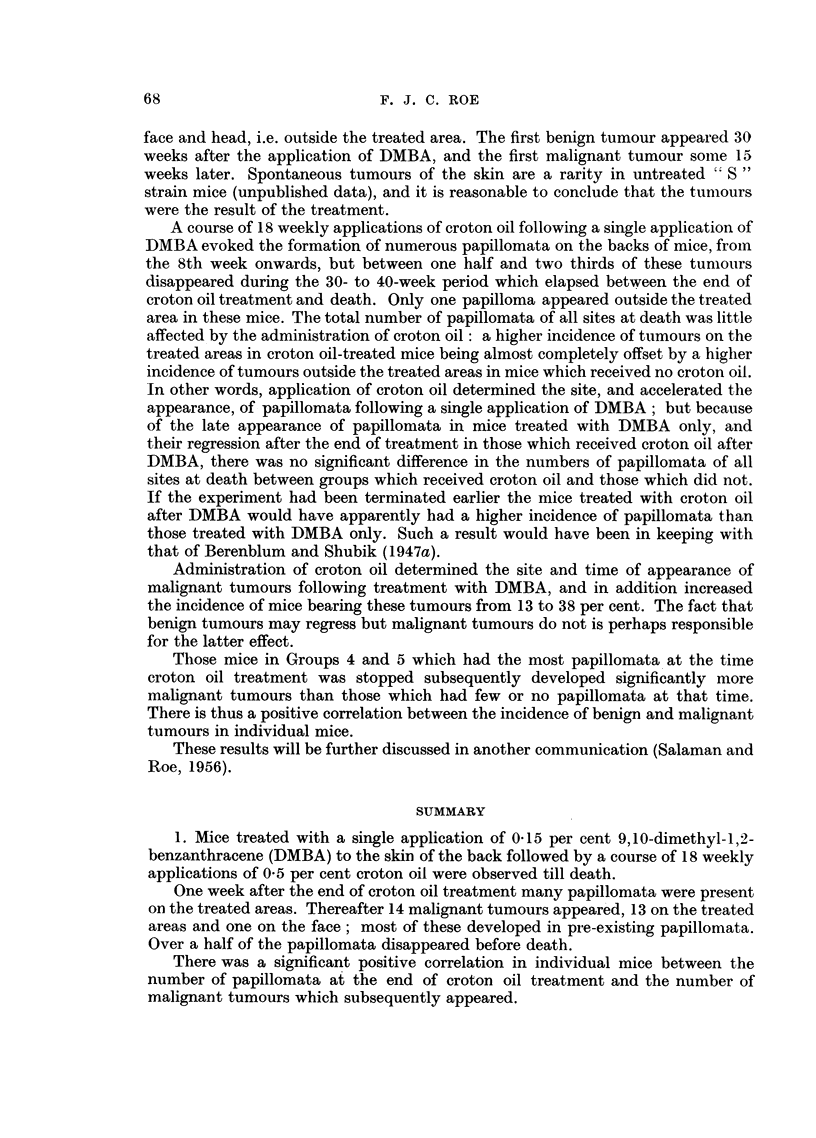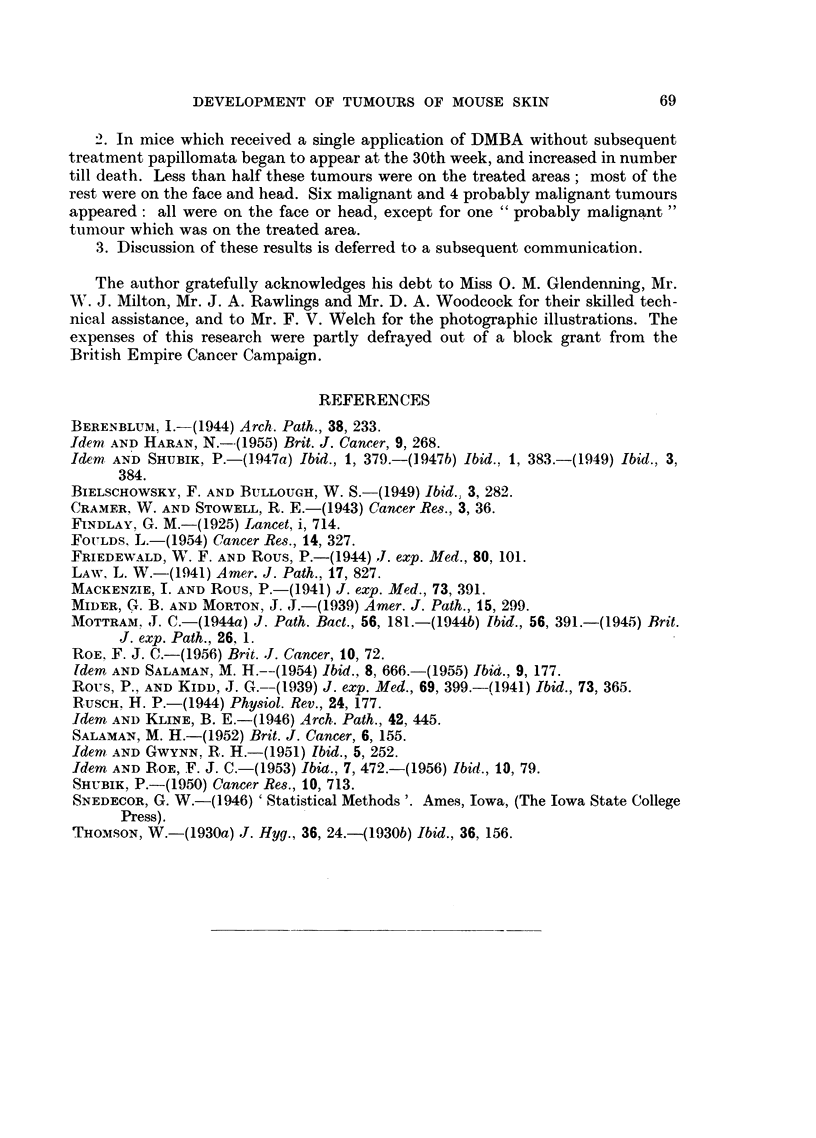# The Development of Malignant Tumours of Mouse Skin after “Initiating” and “Promoting” Stimuli. I. The Effect of a Single Application of 9, 10-Dimethyl-1, 2-Benzanthracene (DMBA), with and without Subsequent Treatment with Croton Oil

**DOI:** 10.1038/bjc.1956.9

**Published:** 1956-03

**Authors:** F. J. C. Roe

## Abstract

**Images:**


### THE DEVELOPMENT OF MALIGNANT TUMOURS OF MOUSE SKIN AFTER " INITIATING " AND " PROMOTING " STIMULI

### I. THE EFFECT OF A SINGLE APPLICATION OF 9,10-DIMETHYL-1,2-BENZANTHRACENE (DMBA), WITH AND WITHOUT SUBSEQUENT TREATMENT WITH CROTON OIL

F. J. C. ROE

*From the Cancer Research Department, London Hospital Medical College, London, E.1*

Received for publication January 19, 1956

MUCH experimental work has been reported which supports the view that carcinogenesis is divisible into stages. For two of these the terms " initiation " and " promotion ", proposed by Friedewald and Rous (1944), have superseded others (for reviews of previous work, and terminologies, see Berenblum, 1944; Rusch, 1944; and Foulds, 1954).

In the past decade many workers in this field have reported the induction of skin tumours in mice by treatment with a single application of a carcinogenic agent (the initiating stimulus) followed by a limited course of croton oil treatment (the promoting stimulus). (Mottram, 1944*a*, 1944*b*, 1945; Rusch and Kline, 1946; Berenblum and Shubik, 1947*a*, 1947*b*, 1949; Bielschowsky and Bullough, 1949; Salaman and Gwynn, 1951; Salaman and Roe, 1953; Berenblum and Haran, 1955). In some of these experiments control mice were given treatment with an initiating dose of the carcinogen alone. Mice so treated developed few or no tumours during a period of observation limited, as a rule, to some 20 to 30 weeks. In other experiments no such control group was included. Similarly, in experiments where control mice were given treatment with croton oil alone for a limited period, few or no tumours developed; but again in several cases this control was omitted.

It is, however, well established that skin tumours may arise after a single application of a carcinogenic agent (Findlay, 1925; Mider and Morton, 1939; Law, 1941; Cramer and Stowell, 1943; Bielschowsky and Bullough, 1949), though usually only after a long latent interval (Findlay, 1925). But it has never been established what proportion of tumours, benign or malignant, appearing in mouse skin after initiating and promoting stimuli would have arisen in the absence of the latter.

Another phenomenon which has been insufficiently considered in work on initiation and promotion is that of regression of tumours. There is no doubt that of the tumours which appear on mouse skin after a single application of 9,10-dimethyl-1,2-benzanthracene (DMBA) followed by a limited course of croton oil treatment, a considerable proportion regress (Shubik, 1950). Similar findings were reported for the rabbit's ear, after various initiating and promoting stimuli, by Rous and his colleagues (Rous and Kidd, 1939, 1941; Mackenzie and Rous, 1941; Friedewald and Rous, 1944).

In this series of four papers the roles of initiator and promoter in the induction of benign and malignant tumours of mouse skin will be reconsidered, using DMBA as the initiating agent and croton oil as the promoting agent.

In the present paper an attempt is made to answer the question whether a limited course of croton oil treatment increases the final incidence of benign and malignant tumours which follow a single application of DMBA, when observation is prolonged till death.

### MATERIALS AND METHODS

*Mice.*—Stock albino male mice of the " S " strain (Salaman and Gwynn, 1951 ; Salaman and Roe, 1953; Roe and Salaman, 1954, 1955) were used throughout. They were fed on cubes prepared according to the Rowett Institute formula (Thomson, 1930*a*, 1930*b*), plus fresh greenstuff twice a week and water *ad libitum.*

Before the experiment was begun mice were vaccinated on the tail with sheep lymph as a precaution against ectromelia : only positive reactors were used.

The age of mice at the beginning of the experiments was between 7 and 9 weeks.

*Chemical agents and solvents.*—9,10-Dimethyl-1,2-benzanthracene (DMBA) was obtained from Messrs. L. Light and Co.

The croton oil used was of the same batch used in previous experiments in this laboratory (Salaman and Gwynn, 1951 ; Salaman and Roe, 1953 ; Roe and Salaman, 1954, 1955). In the third paper of this series (Roe, 1956) it is referred to as Batch I. It was prepared from the seeds of *Croton tiglium* by simple expression, by Messrs. Stafford Allen and Sons, Ltd., 20 Wharf Road, London, N.1.

Acetone was the solvent both for DMBA and croton oil.

*Technique of application.*—The hair of the whole back from forelimbs to tail was clipped at the beginning of the experiment and subsequently when necessary. The solutions were delivered from calibrated pipettes, care being taken that they spread as evenly as possible over the clipped area. 0·2 ml. of the DMBA solution, and 0·3 ml. of the croton oil solution, were applied.

*Histological examination.*—Biopsy specimens and tissues taken for section during post mortem examination were fixed in Zenker's fluid, embedded in paraffin wax, and stained with haematoxylin and eosin-Biebrich scarlet (Salaman and Gwynn, 1951).

### EXPERIMENTAL

Five groups of male mice were used in the experiment. The groups were derived from two batches : A and B. Batch B was received from the animal-breeder approximately three months after Batch A. The age of mice at the beginning of treatment was always between 7 and 9 weeks. Table I shows from

TABLE I.—*Treatment of Groups.*

| Group. | Batch of mice. | Number of mice. | Primary treatment. | Interval. | Secondary treatment. |
|---|---|---|---|---|---|
| 1 | A | 30 | Single application of 0·2 ml. 0·15 per cent DMBA in acetone | — | None |
| 2 | A | 20 | Ditto | — | " |
| 3 | B | 10 | " | — | " |
| 4 | A | 20 | " | 4 weeks | 18 weekly applications of 0·3 ml. 0·5 per cent croton oil in acetone |
| 5 | B | 10 | " | " | Ditto |

TABLE II.—*Incidence of Papillomata.*

| Group. | Number of mice at beginning of experiment. | Number of mice which survived for 36 weeks or more from the beginning of the experiment.* | Average survival from the beginning of experiment of mice shown in previous column (weeks). | Incidence of papillomata at the end of croton oil treatment (22nd week).†: Number of tumour-bearing mice. | Incidence of papillomata at the end of croton oil treatment (22nd week).†: Mean number of tumours per surviving mouse, with standard deviation. | Incidence of papillomata at death.: Total number of tumour-bearing mice. | Incidence of papillomata at death. Mean number of papillomata per mouse, with standard deviation.: On back. | On face and head. | On belly and limbs. | Total on all sites. |
|---|---|---|---|---|---|---|---|---|---|---|
| 1 | 30 | 22 | 67·1 | — | — | 12 | 0·7±0·3 | 0·8±0·3 | 0·2±0·1 | 1·7±0·6 |
| 2 | 20 | 17 | 57·6 | — | — | 12 | 1·3±0·8 | 2·9±0·9 | 0·8±0·4 | 5·0±1·8 |
| 3 | 10 | 8 | 68·7 | — | — | 5 | 0 | 1·4±1·2 | 0·6±0·4 | 2·0±1·2 |
| 4 | 20 | 14 | 56·3 | 11 | 7·1±2·6 | 10 | 1·9±0·7 | 0·1±0·1 | 0 | 2·0±0·8 |
| 5 | 10 | 7 | 59·6 | 6 | 5·6±2·9 | 5 | 2·9±1·7 | 0 | 0 | 2·9±1·7 |

* The figures in this column exclude a few mice in which advanced post-mortem changes rendered tumours uncountable (see text, p. 64). All subsequent columns refer only to the mice listed in this column.

† These figures refer to papillomata on the treated area. Tumours elsewhere did not appear until the 30th week (see text, p. 65).

TABLE III.—*Incidence of Malignant Skin Tumours.*

| Group. | Number of mice at beginning of experiment. | Number of mice which survived for 36 weeks or more from the beginning of the experiment.* | Average survival from beginning of experiment of mice shown in previous column (weeks). | Number of mice bearing malignant tumours.: On back. | On face and head. | Number of malignant tumours.: On back. | On face and head. | Mean time of appearance of malignant tumours (weeks from the beginning of experiment, with standard deviation).: On back. | On face. | Number of "probably malignant" tumours.†: On back. | On face and head. |
|---|---|---|---|---|---|---|---|---|---|---|---|
| 1 | 30 | 22 | 67·1 | 0 | 2 | 0 | 2 | — | 55·3±4·7 | 0 | 1 |
| 2 | 20 | 17 | 57·6 | 0 | 4 | 0 | 4 | | | 0 | 1 |
| 3 | 10 | 8 | 68·7 | 0 | 0 | 0 | 0 | | | 1 | 1 |
| 4 | 20 | 14 | 56·3 | 4 | 0 | 7 | 0 | 46·3±2·5 | 53 | 0 | 0 |
| 5 | 10 | 7 | 59·6 | 3 | 1 | 6 | 1 | | | 0 | 0 |

* The figures in this column exclude a few mice in which advanced post-mortem changes rendered tumours uncountable (see text, p. 64).

† Tumours showing infiltration of the dermis but not of the panniculus carnosus.

which batch mice of the different groups were derived, and the number of mice in each group.

After successful vaccination on the tails with sheep lymph, and removal of hair from the backs by clipping, all the mice were given a single application of 0·2 ml. 0·15 per cent DMBA in acetone. Groups 1, 2, and 3 received no further treatment. Groups 4 and 5 began a course of 18 weekly applications of 0·3 ml. 0·5 per cent croton oil in acetone 4 weeks after the application of DMBA.

During the course of croton oil treatment mice in Groups 4 and 5 were examined weekly for papillomata; after the end of treatment weekly examination was continued, but only malignant tumours were recorded. Mice of Groups 1, 2, and 3 were examined weekly throughout the experiment for the presence of malignant tumours. When a mouse died, or was killed, a record was made of all benign and malignant skin tumours, including any outside the treated area. Occasionally, because the death of a mouse went unnoticed, skin tumours were rendered uncountable by advanced post-mortem changes. Such mice were few, and were omitted from the results recorded in Tables II and III.

If, at the weekly inspection, a mouse was thought to have a malignant tumour of the skin, it was set aside for more frequent observation. When the naked-eye appearance of a tumour left no doubt that it was malignant, and it appeared operable, an attempt was made to remove it by biopsy under ether anaesthesia. The naked-eye assessment of malignancy was based on the following characteristics: ulceration, haemorrhage, an undermined edge, rapid expansion between successive observations, adherence to deep structures, and enlargement of the regional lymph node. Successful operative removal of such tumours made it possible for individual mice to develop multiple malignant tumours in sequence. Careful note was made of the position of scars, lest a local recurrence should be mistaken for a second malignant tumour.

All apparently malignant, and many apparently benign, tumours were taken for histological examination. In addition regional lymph glands were taken for section from mice bearing (or which had borne before operative removal) apparently malignant tumours.

---

### EXPLANATION OF PLATES

Fig. 1.—Carcinoma below the right eye of a mouse 48 weeks after a single application of 9,10-dimethyl-1,2-benzanthracene (DMBA) to the skin of the back. There were also 2 papillomata in the submandibular region: one of these can be seen in the photograph. × 3.

Fig. 2.—Section of tumour shown in Fig. 1. Masses of carcinoma cells have infiltrated the subcutaneous tissues, and in one place have surrounded a nerve deep to a facial muscle. On the right is the large follicle of a vibrissa. × 20.

Fig. 3 and 4.—Medium-power views of two areas in Fig. 2. Fig. 3 shows carcinomatous masses containing a few horny pearls; and Fig. 4, a nerve surrounded by a mass of carcinoma cells deep to facial muscle. × 50.

Fig. 5.—" Probably malignant " tumour (see text, p. 66) below the left eye of a mouse 48 weeks after a single application of DMBA to the skin of the back (Group 1). Note the proximity of this tumour to the follicles of the vibrissae. × 3.

Fig. 6.—Section of a tumour in a mouse which had received one application of DMBA, followed by 18 weekly applications of croton oil. This tumour appeared malignant when first seen, 41 weeks after the beginning of treatment. A carcinomatous mass is seen infiltrating the panniculus carnosus. × 20.

1

5

2

3

4

6

### RESULTS

(a) *The incidence of papillomata.*

In Groups 4 and 5 papillomata began to appear on the backs 8 weeks after the beginning of croton oil treatment, and increased in total number until the end of treatment, though the weekly records indicate quite clearly that some tumours disappeared during this period. Thereafter the total number of papillomata on the backs gradually declined, though, as noted by Salaman (1952), a few new ones appeared.

In Groups 1, 2, and 3 papillomata began to appear during the 30th week of the experiment. Most of these tumours were situated on the face or head, and only few on other sites (backs, bellies, and limbs). The cheeks near the follicles of the vibrissae, the lips, the eyelids, the ears, and the mandibular region were the most commonly affected sites. Not all these tumours persisted until death. Throughout the period of observation tumours of sites other than the back were more numerous in Groups 1, 2, and 3 than in Groups 4 and 5.

Table II shows the numbers of papilloma-bearing mice, and the mean numbers of tumours per surviving mouse, at the end of croton oil treatment and at death. Standard deviations about the means are also given. The mean numbers of tumours on the backs of mice of Groups 4 and 5 at the time of death (i.e. about 60 weeks after the beginning of treatment) were between one-third and one-half the corresponding values at the end of croton oil treatment. In other words, between one-half and two-thirds of the papillomata originally present had disappeared. It will be noted that this regression of tumours after the end of treatment occurred in mice of both batches, A and B.

The mean number of papillomata per mouse *at the end of croton oil treatment* in Groups 4 and 5 exceeded that attained at any time by mice of Groups 1, 2, and 3 ; but the difference was significant only when tumours on the treated areas alone were considered.

The mean numbers of papillomata of all sites *at death* did not differ significantly between groups which had received croton oil treatment and those which had not.

In considering the relation of these results to those of Berenblum and Shubik, it is necessary to bear in mind that the effect on papilloma-incidence of subsequent croton oil applications in mice treated with a single application of DMBA is clearly dependent on the time at which the observations are made. After the end of croton oil treatment the numbers of papillomata in Groups 4 and 5 gradually declined, but those in Groups 1, 2, and 3, which received no croton oil, gradually rose during the corresponding period. It would have been possible, by killing the mice after an arbitrary period shorter than the average life-span, to show an apparent positive influence of croton oil on papilloma-incidence at death similar to that observed by Berenblum and Shubik. Conversely, if the mice had lived longer than they did, and the observed trends had continued, croton oil treatment might have appeared to have had a negative influence. Under the actual conditions of this experiment, however, the most clearly marked difference at the time of death between mice treated with DMBA alone and those treated with DMBA followed by croton oil was not in the incidence of papillomata but in their site : in mice treated with DMBA alone the majority of papillomata were on the faces and heads, whereas in those treated with DMBA and croton oil almost all the tumours were situated on the backs. Mice of both batches (A and B) showed this difference in site of tumours at death.

(b) *Incidence of malignant tumours*

The histological criterion of malignancy for tumours on the back was infiltration of the continuous layer of muscle situated on the deep surface of the dermis, namely, the *panniculus carnosus*. For tumours on the face, infiltration of the facial muscles was the criterion. A few tumours showed other indications of malignancy (e.g. ulceration, anaplasia, and infiltration of the dermal collagen), but failed to fulfil the chosen criterion ; these were called " probably malignant ".

Table III shows the numbers of mice bearing malignant tumours, and the numbers, sites, and mean time of appearance, of malignant tumours in the different groups. The first malignant tumour to appear was in a mouse of Group 4 during the 36th week after treatment with DMBA. In all groups mice which died or were killed before the 36th week were excluded from the table. As stated above, a few mice could not be examined satisfactorily for tumours at death because of advanced post-mortem changes ; these mice were also excluded. The time of appearance of a malignant tumour was arrived at retrospectively : when a tumour was found to be malignant on histological examination the weekly records immediately prior to the date of biopsy or death were consulted, and the date when the tumour was first thought to be malignant on naked-eye examination was taken to be its time of appearance. The incidence and sites of " probably malignant " tumours in the different groups is shown in the last column of the Table.

Among the 47 survivors of Groups 1, 2, and 3 (DMBA only), 6 bore malignant and 4 " probably malignant " tumours. No mouse bore more than one such tumour, nine of which were on the face or head ; the remaining tumour, a " probably malignant " one, was in the scapular region. The mean latent interval between treatment with DMBA and the appearance of the definitely malignant tumours was 55·3 weeks with a standard deviation of $\pm$ 4·7 weeks. Fig. 1–4 show one of the malignant tumours of the face as it appeared to the naked eye and microscopically. Fig. 5 shows the naked-eye appearance of a " probably malignant" tumour of the face.

Among the 21 survivors of Groups 4 and 5 (DMBA followed by croton oil), 13 malignant tumours appeared on the back and 1 on the face. There were no " probably malignant " tumours in these groups. Altogether 8 mice bore malignant tumours ; 1 bore 3 tumours, 4 bore 2 tumours, and 3 bore one tumour each. The mouse with the facial tumour had no other malignant tumours. The mean latent interval between treatment with DMBA and the appearance of malignant tumours on the back was 46·3 weeks with a standard deviation of $\pm$ 2·5 weeks. The facial tumour appeared after an interval of 53 weeks.

All the malignant tumours were of epithelial origin. All grades from well-differentiated squamous-cell carcinomata to highly anaplastic tumours were encountered.

Of the mice bearing definitely malignant tumours in Groups 1, 2, and 3, two had metastases in the lymph glands of the neck. One of the mice in Group 4, and 1 in Group 5, bearing malignant tumours on the back, had metastases in the regional (i.e. axillary or inguinal) lymph glands. No metastases at more distant sites were found.

There was evidence, either from the preceding weekly records, or from the histological appearance, that the majority of malignant tumours arose in pre-existing papillomata. A few, however, appeared malignant from the time they were first seen. Fig. 6 shows a section of such a tumour.

(c) *Relation between incidence of papillomata and carcinomata*

The results suggested that there was a relation between the site of occurrence of papillomata and carcinomata. Groups 1, 2, and 3, which had most papillomata on the face or head at death, also had most carcinomata on this site. Similarly, Groups 4 and 5, which had most papillomata on the backs, also had most malignant tumours on this site.

The correlation coefficient ($r$) between the number of papillomata and the number of carcinomata, on individual mice, was calculated (Snedecor, 1946, p. 138). Values of $r$ between 0 and $+ 1 \cdot 0$ indicate a positive correlation between the variates, and values between 0 and $- 1 \cdot 0$ a negative correlation. When $r$ is zero the two variates are uncorrelated. The probability ($P$) that a correlation between the two variates, at least as big as the observed value, would have occurred by chance is given in tables for $r$ under the heading of the appropriate number of degrees of freedom ($n - 2$, where $n$ is the number of mice).

Firstly a correlation table was prepared for mice of Groups 1, 2, and 3. In this case the two variates were the number of benign tumours, and the number of malignant tumours, on the face and head of individual mice at death. The calculated value for $r$ in this test was $+ 0 \cdot 09$ on 45 degrees of freedom. This slightly positive correlation between the two variates did not reach the customary 5 per cent level of significance.

In a second test the two variates were the number of papillomata on the backs of individual mice of Groups 4 and 5, *at the end of croton oil treatment*, and the number of definitely malignant tumours which subsequently arose on the backs of the same mice. The calculated value for $r$ was $+ 0 \cdot 57$ on 19 degrees of freedom, and the probability ($P$) that this positive correlation would have occurred by chance was less than 1 per cent.

A final test was made for correlation between the number of papillomata on individual mice of Groups 4 and 5 *at death*, and the total number of malignant tumours on the same mice. Again, a significantly positive correlation was found ($r = + 0 \cdot 465$ on 19 degrees of freedom, $0 \cdot 01 < P < 0 \cdot 05$).

An observation reported by Shubik (1950) is relevant at this point : he stated that when a mouse developed a malignant tumour of the skin, many of its benign tumours regressed. In the present series (Groups 4 and 5) the regression rate of papillomata following the end of croton oil treatment was not significantly different in mice which developed malignant tumours and in those which did not, namely 56 per cent in the former and 57·5 per cent in the latter. The numbers of animals in this series was small for such a comparison, but in a similar and much larger series (to be described in a later paper, Salaman and Roe, 1956) the papilloma-regression rate was actually higher in mice without malignant tumours than in those with them, namely 17 per cent and 10 per cent, respectively. (The period between the end of treatment and death was considerably shorter in this case.) There was virtually no difference in the average survival-time between the two classes in either of these series.

### CONCLUSIONS

After a single application of 0·2 ml. 0·15 per cent DMBA to the backs of mice of the " S " strain, there appeared many benign and a few malignant tumours. The majority of the benign, and all the malignant, tumours arose from the skin of the face and head, i.e. outside the treated area. The first benign tumour appeared 30 weeks after the application of DMBA, and the first malignant tumour some 15 weeks later. Spontaneous tumours of the skin are a rarity in untreated "S" strain mice (unpublished data), and it is reasonable to conclude that the tumours were the result of the treatment.

A course of 18 weekly applications of croton oil following a single application of DMBA evoked the formation of numerous papillomata on the backs of mice, from the 8th week onwards, but between one half and two thirds of these tumours disappeared during the 30- to 40-week period which elapsed between the end of croton oil treatment and death. Only one papilloma appeared outside the treated area in these mice. The total number of papillomata of all sites at death was little affected by the administration of croton oil : a higher incidence of tumours on the treated areas in croton oil-treated mice being almost completely offset by a higher incidence of tumours outside the treated areas in mice which received no croton oil. In other words, application of croton oil determined the site, and accelerated the appearance, of papillomata following a single application of DMBA ; but because of the late appearance of papillomata in mice treated with DMBA only, and their regression after the end of treatment in those which received croton oil after DMBA, there was no significant difference in the numbers of papillomata of all sites at death between groups which received croton oil and those which did not. If the experiment had been terminated earlier the mice treated with croton oil after DMBA would have apparently had a higher incidence of papillomata than those treated with DMBA only. Such a result would have been in keeping with that of Berenblum and Shubik (1947*a*).

Administration of croton oil determined the site and time of appearance of malignant tumours following treatment with DMBA, and in addition increased the incidence of mice bearing these tumours from 13 to 38 per cent. The fact that benign tumours may regress but malignant tumours do not is perhaps responsible for the latter effect.

Those mice in Groups 4 and 5 which had the most papillomata at the time croton oil treatment was stopped subsequently developed significantly more malignant tumours than those which had few or no papillomata at that time. There is thus a positive correlation between the incidence of benign and malignant tumours in individual mice.

These results will be further discussed in another communication (Salaman and Roe, 1956).

### SUMMARY

1. Mice treated with a single application of 0·15 per cent 9,10-dimethyl-1,2-benzanthracene (DMBA) to the skin of the back followed by a course of 18 weekly applications of 0·5 per cent croton oil were observed till death.

One week after the end of croton oil treatment many papillomata were present on the treated areas. Thereafter 14 malignant tumours appeared, 13 on the treated areas and one on the face ; most of these developed in pre-existing papillomata. Over a half of the papillomata disappeared before death.

There was a significant positive correlation in individual mice between the number of papillomata at the end of croton oil treatment and the number of malignant tumours which subsequently appeared.

2. In mice which received a single application of DMBA without subsequent treatment papillomata began to appear at the 30th week, and increased in number till death. Less than half these tumours were on the treated areas ; most of the rest were on the face and head. Six malignant and 4 probably malignant tumours appeared : all were on the face or head, except for one " probably malignant " tumour which was on the treated area.

3. Discussion of these results is deferred to a subsequent communication.

The author gratefully acknowledges his debt to Miss O. M. Glendenning, Mr. W. J. Milton, Mr. J. A. Rawlings and Mr. D. A. Woodcock for their skilled technical assistance, and to Mr. F. V. Welch for the photographic illustrations. The expenses of this research were partly defrayed out of a block grant from the British Empire Cancer Campaign.

### REFERENCES

BERENBLUM, I.—(1944) *Arch. Path.*, **38**, 233.

*Idem* AND HARAN, N.—(1955) *Brit. J. Cancer*, **9**, 268.

*Idem* AND SHUBIK, P.—(1947*a*) *Ibid.*, **1**, 379.—(1947*b*) *Ibid.*, **1**, 383.—(1949) *Ibid.*, **3**, 384.

BIELSCHOWSKY, F. AND BULLOUGH, W. S.—(1949) *Ibid.*, **3**, 282.

CRAMER, W. AND STOWELL, R. E.—(1943) *Cancer Res.*, **3**, 36.

FINDLAY, G. M.—(1925) *Lancet*, i, 714.

FOULDS, L.—(1954) *Cancer Res.*, **14**, 327.

FRIEDEWALD, W. F. AND ROUS, P.—(1944) *J. exp. Med.*, **80**, 101.

LAW, L. W.—(1941) *Amer. J. Path.*, **17**, 827.

MACKENZIE, I. AND ROUS, P.—(1941) *J. exp. Med.*, **73**, 391.

MIDER, G. B. AND MORTON, J. J.—(1939) *Amer. J. Path.*, **15**, 299.

MOTTRAM, J. C.—(1944*a*) *J. Path. Bact.*, **56**, 181.—(1944*b*) *Ibid.*, **56**, 391.—(1945) *Brit. J. exp. Path.*, **26**, 1.

ROE, F. J. C.—(1956) *Brit. J. Cancer*, **10**, 72.

*Idem* AND SALAMAN, M. H.—(1954) *Ibid.*, **8**, 666.—(1955) *Ibid.*, **9**, 177.

ROUS, P., AND KIDD, J. G.—(1939) *J. exp. Med.*, **69**, 399.—(1941) *Ibid.*, **73**, 365.

RUSCH, H. P.—(1944) *Physiol. Rev.*, **24**, 177.

*Idem* AND KLINE, B. E.—(1946) *Arch. Path.*, **42**, 445.

SALAMAN, M. H.—(1952) *Brit. J. Cancer*, **6**, 155.

*Idem* AND GWYNN, R. H.—(1951) *Ibid.*, **5**, 252.

*Idem* AND ROE, F. J. C.—(1953) *Ibid.*, **7**, 472.—(1956) *Ibid.*, **10**, 79.

SHUBIK, P.—(1950) *Cancer Res.*, **10**, 713.

SNEDECOR, G. W.—(1946) ' Statistical Methods '. Ames, Iowa, (The Iowa State College Press).

THOMSON, W.—(1930*a*) *J. Hyg.*, **36**, 24.—(1930*b*) *Ibid.*, **36**, 156.